# Framing of COVID-19 in Newspapers: A Perspective from the US-Mexico Border

**DOI:** 10.3390/healthcare10122362

**Published:** 2022-11-24

**Authors:** Rifat Afrin, Ahasan Harun, Gayle Prybutok, Victor Prybutok

**Affiliations:** 1 Department of Rehabilitation and Health Services, College of Health and Public Service, University of North Texas, Denton, TX 76203, USA; 2 Department of Information Systems, Robert C. Vackar College of Business & Entrepreneurship, University of Texas-Rio Grande Valley, Edinburg, TX 78539, USA; 3 Department of Information Technology and Decision Sciences, G. Brint Ryan College of Business, University of North Texas, Denton, TX 76203, USA

**Keywords:** newspapers, COVID-19, health communication, community health

## Abstract

The degree to which the media report a health emergency affects the seriousness with which the people respond to combat the health crisis. Engagement from local newspapers in the US has received scant scrutiny, even though there is a sizable body of scholarship on the analysis of COVID-19 news. We fill this void by focusing on the Rio Grande Valley area of the US-Mexico border. To understand the differences, we compared such local news coverage with the coverage of a national news outlet. After collecting the relevant news articles, we used sentiment analysis, rapid automatic keyword extraction (RAKE), and co-occurrence network analysis to examine the main themes and sentiments of COVID-19 news articles. The RAKE identified that county-specific news or local regulations are more prevalent among the key terms in The Monitor which are absent in USA Today. The co-occurrence network shows the coverage of the disruption of sports season in USA Today which is not present in The Monitor. The sentiment analysis presents fear emotion is more dominant in USA Today, but trust emotion becomes more prevalent in The Monitor news coverage. These findings show us that, although the subject of the health emergency is the same, local and national newspapers describe it in different ways, and the sentiments they convey are also not the same.

## 1. Introduction

Over the past few decades, outbreaks of infectious diseases have become more common [1]. Efficient risk management depends on people’s desire to learn about diseases and take precautions to stop them from propagating [2]. Against the backdrop of a disease outbreak, a knowledgeable and involved public is essential for maintaining public health [3]. Prior research has explored how journalistic creativity is sparked during such emergencies [4]. However, given its vast reach, the absence of a disaster epicenter, harm to human lives, and the capacity to inflict damage across the bulk of capitalistic sectors, the COVID-19 pandemic posed a unique confluence of these factors. Therefore, effective communication through news media became necessary to maintain public awareness. This study provides a perspective on how health emergencies are covered in local newspapers of the Rio Grande Valley area of the US-Mexico border.

To understand how news and public opinion influence one another, we can resort to news agenda-setting theory [5]. One of the basic tenets of agenda-setting theory is that the public values the same things that the media consider significant or conspicuous [6]. The media has an impact on what topics people perceive to be relevant or significant. McCombs and Shaw [7] created and popularized the hypothesis fifty years ago (1972). Since then, a great deal of empirical and theoretical research has been performed, and it has undergone numerous adaptations. The idea of second-level or attribute agenda-setting stands out among them [8,9], which states that media impact not only what the audience considers to be important but also what the majority think about a topic or some of its attributes.

It may be argued that the emergence of social media has changed the agenda-setting function of traditional media [10]. However, print news reporting continues to serve as the main source for the majority of what is later reported in other media, engaging a wider audience than just newspaper readers [11]

A health emergency’s level of media coverage impacts how seriously people take it, which could have a big impact on what they do to deal with the issue. Prior research has examined how scientific and medical issues are presented in the media, focusing on how infectious diseases are reported in news updates (e.g., [12,13]). However, we still lack knowledge about how health crises are reported in the press, specifically whether there are distinctions between local and national reporting in media news frames. 

As long as they remain key sources of information for the general public, the media will continue to have a significant impact on how individuals perceive and respond to threats [14]. Instead of just disregarding press coverage, it is crucial to understand how such tales are generated and delivered in certain situations to significantly influence communication and management actions [14]. The highest readership and influence levels of any media may be found in newspapers in the United States [15]. For this reason, it is crucial to examine how a pandemic emergency like COVID-19 was covered by the newspapers to learn how to prepare for similar situations in the future.

In a journalist’s conscientious pursuit of the truth, Ward [16] contends, the field of communication ought to redefine what objectivity means going forward rather than striving to dismiss it entirely. However, despite highlighting the need for trustworthy information, the COVID-19 pandemic has depleted the required resources of local news organizations to accurately narrate the effects of the virus on neighborhood populations [17]. Half (57%) of the 2485 counties in the United States that recorded a COVID-19 infection case in the initial three weeks of this public health emergency either have only one local newspaper or do not have any at all [18]. This study highlights the importance of local newspapers by focusing on how the same COVID-19 pandemic was covered in national and local newspapers.

COVID-19 was undoubtedly one of the most discussed topics in newspapers in 2020. The previous discussion about public health-related topics such as obesity, HPV vaccine deviated from the issues that needed attention and focused more on the debates [19,20]. During the emergence of a health crisis, lots of information flows, and thereby it necessitates a lot of care to handle the information to minimize the risk [21]. In this regard, the question arises of how newspapers are discussing the pandemic topic. In the United States, people read newspapers published by local outlets and national outlets to be informed about emergencies.

Because of the recession, many newspapers are losing advertisement revenues and forced to cut back workers or shutdown. Rio Grande Valley also suffered the loss of two local newspapers (The Edinburg Review and Valley Town Crier) because of the pandemic [22]. Evidence suggests that when a local newspaper closes, civic involvement declines, elected leaders become less responsible, corruption spreads more widely, and voter turnout declines and gets more divisive [23].

How a certain topic is portrayed in the media affects how the public and decision-makers comprehend and interact with it [24] Journalists can shape or alter the audience’s perspective of a crisis. Several ways can be applied to do so: the level of attention the reporters devote to the issue, the emotion and tone used in the news coverage, and how the issue is presented through frames [25]. Since the beginning, the COVID-19 pandemic dominates in major news consumption, and it prompted the President to hold press conferences frequently; but many Americans also depend on their local news outlets to get information regarding the outbreak [26]. According to a survey, approximately 61% of the American adult population are “giving about equal attention to both national and state and local coronavirus news” [27]. Local news has become very important during the COVID-19 outbreak period as several newspapers took initiatives to present “COVID-19 case maps” and zip codes and they also provided details about local business closures and reopening [28]. Therefore, this study was initiated to find how COVID-19 was covered in the early stages of the pandemic in national and local newspapers to gain a perception about future health crisis management.

There is now research going on about future pandemic preparedness [29,30] which implies the US public health needs to be prepared for future pandemics. As news media is an important medium to disseminate crucial information during a health crisis, we need to begin with how COVID-19 was covered in American news media. Prior research has examined how scientific and medical issues are presented in the media, focusing on how infectious disease is reported in news updates (e.g., [12,13]). However, we still lack knowledge about how health crises are reported in the local press, specifically whether there are distinctions between local and national reporting in media news frames. This is a crucial topic because many people depend on local news sources for information. Hence, this tendency to read local news may be reinforced whenever a communicable disease strikes a country’s specific region [2]. As a result, it is necessary to explore how local media cover health issues. Moreover, some individuals may be only curious to read selected newspaper sections, like the international affairs section. Therefore, they prefer only to read major papers because local publications typically do not handle international policy [31] unless that policy affects that area. How can the value of reading local newspapers be conveyed if we do not contrast the frames across different types of newspapers during times of health crisis? Hence, we examine the news headline frames from The Monitor, a reputable local daily from the heavily affected Rio Grande Valley (RGV), and USA Today, a renowned national newspaper.

To fulfill our research goal, we will initially use co-occurrence network analysis of news headlines of the news pieces that mention COVID-19-related key terms between March and April of 2020. Afterward, we will use rapid automatic keyword extraction (RAKE) to determine the most prevalent frames in news headlines. These methods assist in deciphering the key frames that are typically shown in COVID-19 headlines. Then, we will use the sentiment analysis to reveal the types of feelings these headline frames contain and whether they have the potential to cause individuals to become anxious and take protective actions. By nature, the analysis of news headlines is very intricate. A nuanced perception will be elusive if the analysis is made from a single disciplinary viewpoint. Hence, this research will take an interdisciplinary approach. In doing so, our research will shed light on how health professionals and the public may adjust their health responses in reaction to news coverage by concentrating on the key frames and sentiments identified in this study.

Numerous subfields of the psychosocial and health sciences now regularly conduct studies integrating qualitative and quantitative research components [32]. This study also used a combination of qualitative and quantitative methods. For example, an example of a qualitative method is sentiment analysis [33]. Sentiment analysis finds diverse types of emotions such as anger, sadness, or trust from the text documents. However, at the same time these qualitive information also provides us with quantitative information. For example, the trust emotion is greater in local newspaper “The Monitor” articles than in national newspaper USA Today.

Triangulation is a technique for boosting the credibility and validity of study findings. Validity is the degree to which research truly describes or evaluates the notion or concepts being explored. Credibility is the term used to describe the reliability and plausibility of a study [34]. By integrating theories, approaches, and observers in a study, triangulation can assist guarantee that underlying biases brought on by the use of a single approach or a single observation are addressed [35]. 

The fact that data representing real-life experiences are regularly used in mixed method research approaches is a key strength [36]. Because of lockdowns, effects on education and the economy, and family member losses during the pandemic, it is crucial for my study to use a mixed method to capture the essence of human lives. It will be impossible to capture the substance of experiences or reporting quality during a pandemic by employing only qualitative or quantitative methodologies. Thanks to mixed approaches, a research subject can be looked at from many perspectives. For example, one can combine the rich, subjective perspectives on complex situations offered by qualitative inquiry with the generalizable, standardized data from quantitative research. In an effort to provide readers with a more complete explanation, triangulation also makes an effort to examine and describe complex human behavior through a variety of ways. Triangulation is one of the important goals that mixed methods research try to fulfill or look for agreement, confirmation, and connection between the outcomes of several procedures [37].

Co-occurrence network analysis was suggested in earlier research as a valid method for visualizing potential connections between and among different constructs or attributes [38]. As a result, by initially identifying topic distribution and connections as well as prospective connections between topics under study, quantitative researchers may profit from the approach’s results [39]. To locate the keywords, RAKE looks for keyword pairs that are present in the same sequence, at least twice, and together within the same document. A potential candidate term is produced by combining such keywords with their corresponding internal stop words. The score for this new term is the total of its member keywords’ scores [40].

Although traditional framing studies have generally used human coders to identify dominant frames in news articles, the approach is subjective and time-consuming [41,42]. Since the 1990s, when digital technologies first began to take off, a lot of news content has accumulated, necessitating automated frame detection [43]. A group of machine learning algorithms known as automated content analysis are used often in medical research and social sciences and to synthesize both qualitative and quantitative data from large bodies of text [44]. Or in other words, we can obtain both qualitative and quantitative information from the techniques of automated content analysis.

## 2. Background

Studying the initial period of pandemic is necessary because it provides us insight into communication tactics during the early period of a health emergency. Public health officials communicated preventive measures for minimizing the rates of infections. News media organizations also began to rush over to cover COVID-19 by emphasizing various facets of the pandemic. Hence, it is necessary to study news frames from this initial stage. Because news media frames are not only about shaping public opinion, but news media frames also work as a medium of communication to let the public know about pandemic management interventions [45].

The context is now very different now in 2022. However, health emergency situations like COVID-19 can happen in the future. Therefore, we need to look back to building communication strategies for future health emergencies so that the situation will not turn more severe like COVID-19 and the public and health officials can be more prepared. To improve early pandemic management techniques, researchers need to consider how the media report on health crises at their earliest stages.

Willacy, Hidalgo, Starr, and Cameron counties comprise the RGV’s four counties bordering Mexico. Contrary to the entire Texas state, Texas’ bordering counties have unique attributes [46]. There is a need for culturally sensitive approaches to increase the outreach to this demographic [47]. The prevalence and fatality rates for cervical cancer are higher in the RGV compared to other regions of Texas and to the remainder of the United States. Notably, compared to women residing in other regions of Texas or the rest of the nation, women in RGV have a 30% greater cervical cancer frequency with associated fatalities [46]. This region is also especially susceptible to HIV infection due to the enormous number of people passing through its border crossing points and the economic hardship that individuals and neighborhoods must endure [48]. Because of increased poverty, low rates of health insurance coverage, and significant numbers of immigrants from Latin America during a period when anti-immigrant stances in the US were on the rise, the RGV was well-positioned to suffer greatly from the coronavirus pandemic [49]. Thus, it makes us curious about how the news media in the RGV portrayed the pandemic and how much it differed from the coverage in a national newspaper.

USA Today is one of the daily U.S. Newspapers with a large readership besides Wall Street Journal and The New York Times [50]. News from the United States and throughout the world is delivered via national newspapers [31]. We can know what is happening in the US and around the globe from USA Today. It can be either political news or sports news or financial news, in other words all types of news are present in a national newspaper to attract the attention of diverse readers. 

The Monitor has the largest readership in Rio Grande Valley (RGV) [51]. This region’s economic and health inequities are among the worst in the country. Most locals are Hispanic, undereducated, and economically underprivileged. They are also underserved in health care and have poor health outcomes [46].

Even before COVID-19, the RGV, situated in deep South Texas, was of interest to many academics and researchers all over the U.S. and major American news media. The RGV’S geographical position at the U.S.-Mexico border, poverty, geopolitics, culture, eco-diversity, trade activities, public health issues, and immigration, etc. are among the topics examined [46,52,53,54,55]. Among public health issues, the RGV gets attention for its higher rates of obesity and diabetes among the people living in colonias [56]. According to research from the Federal Reserve Bank of Dallas, a colonia is a neighborhood along the Texas-Mexico border that could not have access to many of the most basic amenities, such as clean water, sanitary sewers, power, good streets, or safe housing [57]. 

Since the Boston smallpox outbreak of 1721, those responsible for providing the American people with important health information have played two roles: one centered on dissemination of crucial scientific truths, and the other on dismantling lies and misinformation [58]. Considering the need for print, broadcasting, and internet media to inform the public about possible health dangers, the fundamental values of journalism [59] are consistent with the need to report on the pandemic. Nevertheless, people now question the accuracy and authenticity of the information published due to the rush of new information providers populating the digital environment [60]. The need for factual reporting, free of gossip about health issues, is exemplified by outbreak reporting during COVID-19. Newspapers were recognized for providing the public with medical information during the 1918 Spanish flu pandemic [58]. Newspapers reporting COVID-19 continued to educate the people about what precautions to follow, things to avoid, symptoms people should watch out for, and where they can get assistance if afflicted [61].

## 3. Materials and Methods

### 3.1. Data Collection

The news articles were collected using the NewsBank database. NewsBank is a news database resource. Its headquarters is in Florida, USA. The Monitor is selected to represent the RGV. Each day, 40,000 copies of this newspaper are distributed [51]. This is a considerable sum for the RGV. USA Today was selected to represent national newspapers of the United States. From Monday through Friday, USA today is read by nearly one million people daily [62]. To identify news articles, we used search terms that included “COVID-19”, “corona”, “coronavirus”, or “COVID”. These queries yield pertinent details regarding COVID-19 reporting [12]. 566 news for March and April of 2020 were collected from The Monitor. For USA Today, 1159 news items were collected for the same time frame.

### 3.2. Analysis Procedure

We started by using a co-occurrence network method. This co-occurrence network explains the connections among frequently used words. Every word is represented within that figure by a node, and edges show its relationships to specific other keywords. Node sizes are dictated by word frequency, which is likewise relevant for edge size, which is mostly influenced by the Jaccard coefficient. The Jaccard coefficient, which has a value between zero and one, is a statistical measure widely used to assess how similar two things are [63]. Additionally, the study applied the RAKE (rapid automatic keyword extraction) technique. This method for extracting terms or phrases from texts is unsupervised, language- and domain-independent [64]. To extract topics from a text, RAKE creates a list of probable terms from the article’s content. To identify the phrases, RAKE looks for keyword combinations that appear across the same document sources in the same sequence and at least a few times. These words are joined with the stop words that serve as their foundation to create a potentially qualified term. The new keyword’s overall score is calculated by averaging the values of each of its component elements.

Co-occurrence networks are advantageous because they show the network of knowledge elements [65] or make a visual representation to understand the themes in text data. This analysis reveals which themes are essential while also illuminating the connections between the keywords or themes [66]. Therefore, quantitative researchers may benefit from the approach’s findings by initially detecting topic distribution and links as well as potential connections between subjects under study [39]. The co-occurrence network does not assess the rate of word usage because it just illustrates the relationships among topics. Therefore, RAKE is employed to achieve that goal.

The study also carried out an automated sentiment assessment. Empirical evidence shows the significance of sentiments in health-related domains [67]. Due to the enormous amount of unstructured text data that is currently available, sentiment analysis is growing in popularity as a research application area [68]. The method of gathering subjective information about unique sentiments and viewpoints from natural language materials is known as sentiment analysis [69]. A lexicon-based technique was employed for this study. This lexicon is a collection of terms with emotional annotation. Keywords in the data are assigned sentiment ratings based on the results of a lexicon. Regarding communications across various networks, media acts as a paradigm or platform [70]. Hence, it is crucial to comprehend how emotional health and information interact [71]. The sentiment analysis was carried out to emphasize the pandemic’s emotional resonance. Through bar diagrams, we identified the word count for each emotional valence (joy, trust, surprise, sadness, fear, anticipation, disgust, and anger). Moreover, a radar chart (a graph representation for quantifying many dimensions of a phenomenon [72]) and plots have been used to demonstrate each emotion’s top words or key terms. 

## 4. Results

**Figure 1:**
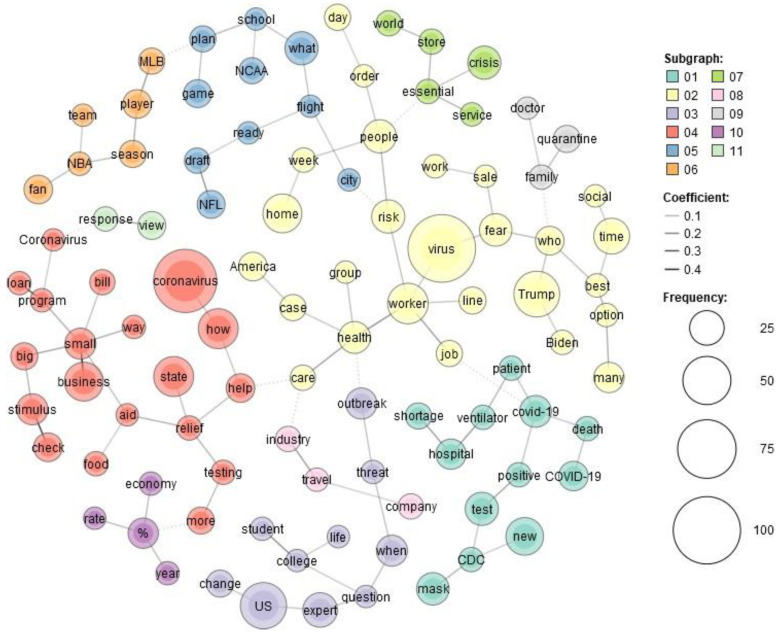
Co-occurrence network for USA Today.

**Figure 2:**
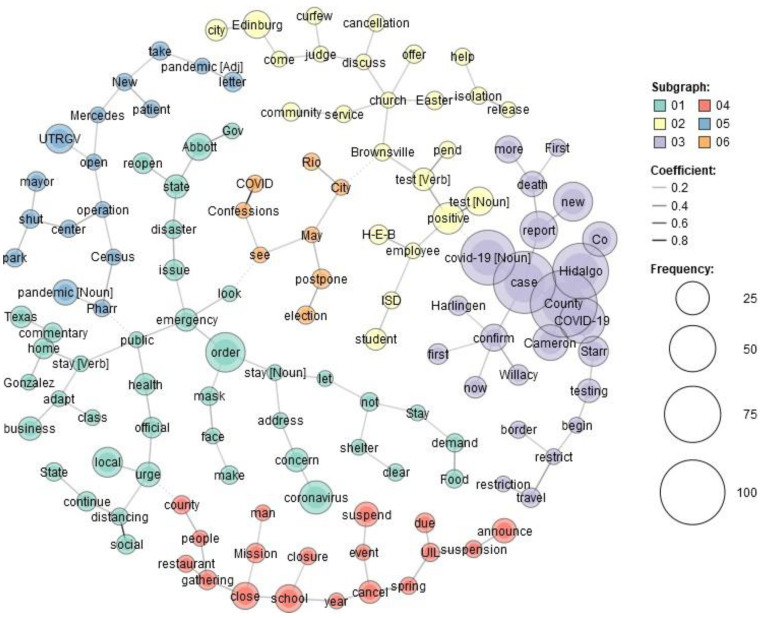
Co-occurrence network for The Monitor.

Figure 1 and Figure 2 show the co-occurrence of the top words in USA Today and The Monitor, respectively.

USA Today contains 11 clusters compared to The Monitor, which has 6. This is not surprising, as USA Today is a large-scale national newspaper. It discusses various themes in more detail than a local newspaper to attract the attention of a wider audience. For USA Today, we can see in frame 1 the keywords CDC, shortage, ventilator, mask, etc. For example, there were supply shortages for health departments. The lack of essential components made testing difficult. Additionally, at the beginning, there were also struggles for ventilators. Frame 2 consists of the virus fear, US national leaders, America, etc. As the experience of this pandemic was still new in the beginning period of March and April of 2020, people were afraid of the virus. In frame 3, we notice the US, expert, question, student, college, etc. This frame explains the threat of the outbreak to different aspects of life, such as education. Experts tried to answer the concerns of both old and young people. Frame 4 provides an idea about the economy during the pandemic as we can see the words loan program, relief aid, small business, etc. For example, customers declined sharply, so small businesses suffered greatly from the pandemic. Both frames 5 and 6 show the disturbance in regular sports events because of the pandemic. Frame 7 shows essentials, service, store, etc. This frame discusses what type of stores or services should be deemed essential. Frame 8 presents the travel industry or travel companies. Because of the travel bans, the travel industry suffered at that time. Frame 9 presents family, doctor, and quarantine. Several families suffered greatly from the pandemic as several members of the same family suffered from the virus. Moreover, the doctors were anxious about keeping their families safe, fearing they would spread the virus to their family members after performing their duties when they returned home. Frame 10 provides more ideas about the economy as it connects with frame four. As Americans began to avoid film theaters or shopping malls, the economy experienced a huge toll. The last frame or frame 11 shows the view and response. Some polls showed the response to the pandemic were mixed views. In other words, their views were partisan or divided across party lines.

In contrast, in The Monitor, we can see the governor and other local leaders, state, local, etc., in frame 1. Student, employee, Community, and two cities of RGV Brownsville, Edinburg are among the words that are present in frame 2. The four counties of the RGV (Willacy, Starr, Hidalgo, Cameron) are present in frame 3. Frame 4 provides an idea about the cancellation of regular gatherings in RGV as we can see the words school, restaurant, cancel, suspend, etc. Frame 5 provides us with more idea about local activities that were hampered because of the pandemic as we see the words shut, operation, Census, etc. Frame 6 provides experiences of the people of COVID as we can see the words COVID, confession, and hindrance in more local activities such as local elections. These frames underlie the notion that national and local newspapers report the same health emergency differently.

**Figure 3:**
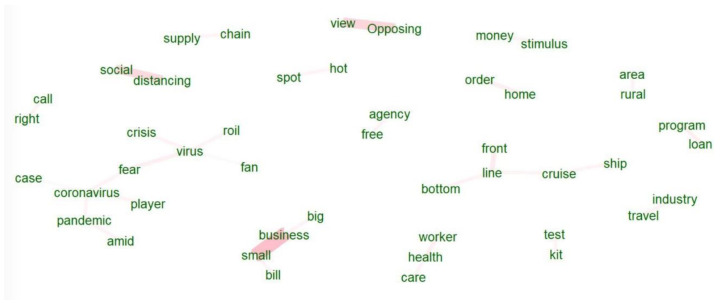
Co-occurrences within three words’ distance: nouns and adjectives (USA Today).

**Figure 4:**
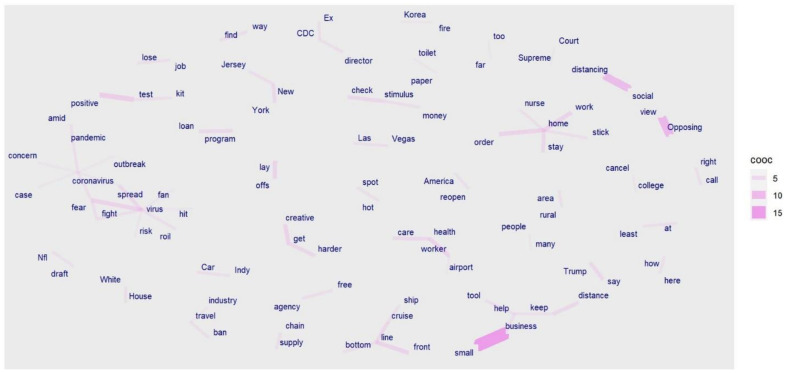
Words following one another (USA Today).

**Figure 5:**
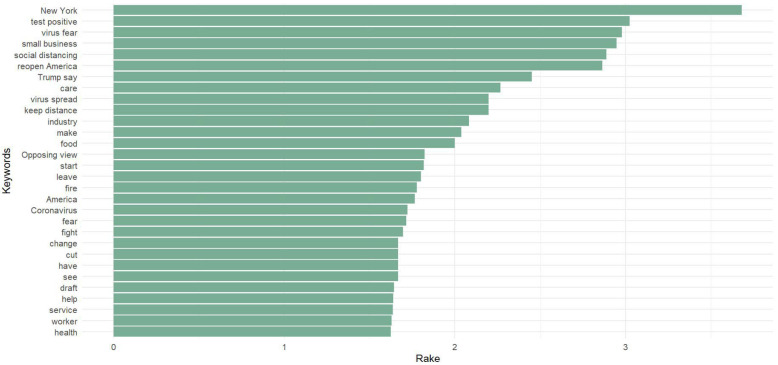
Keywords (noun, pronoun, verb, adjective) identified by RAKE (USA Today).

**Figure 6:**
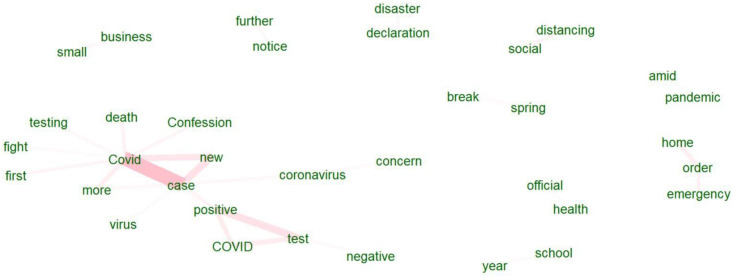
Co-occurrences within three words’ distance: nouns and adjectives (The Monitor).

**Figure 7:**
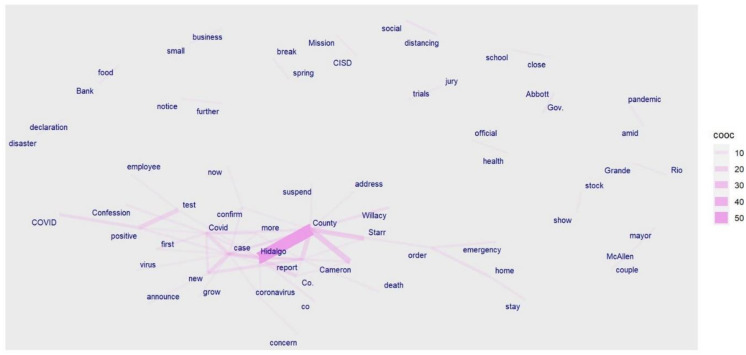
Words following one another (The Monitor).

**Figure 8:**
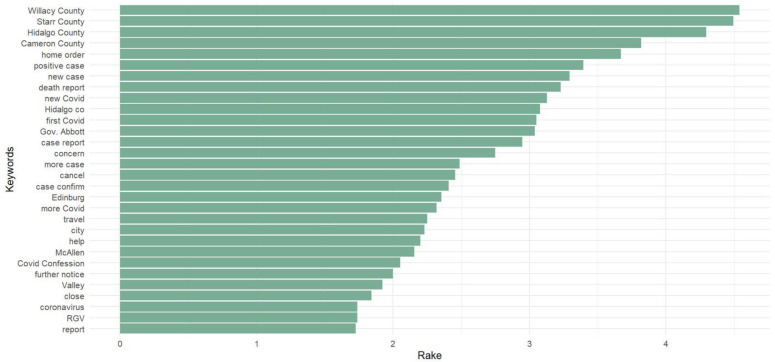
Keywords (noun, pronoun, verb, adjective) identified by RAKE (The Monitor).

We can see more differences in news headline frames in Figure 3, Figure 4, Figure 5, Figure 6, Figure 7 and Figure 8.

Figure 3 and Figure 6 portray the co-occurrence of nouns and adjectives for USA Today and The Monitor, respectively. The travel industry, cruise ships, and stimulus money only appear in USA Today. However, spring break and school year only appear in The Monitor. Moreover, some nouns and adjectives that mostly cooccur together in USA Today are front line, test kit, health care, health worker, rural area, hot spot, the right call, and opposing view. For The Monitor, we can see the nouns and adjectives that mostly cooccur together are emergency order, disaster declaration, further notice, social distancing, COVID confession, etc. Figure 4 and Figure 7 show that words frequently cooccur together and build a frame. These figures differ from Figure 3 and Figure 6 because the previous figures only identify the nouns and adjectives that cooccur together. However, Figure 4 and Figure 7 provide a more extensive viewpoint because words that cooccur together in the two newspapers are displayed here. For USA Today, Figure 4 shows us layoffs, CDC director, New Jersey, New York, Las Vegas, White House, toilet paper, NFL draft, reopen America, stimulus check, job loss, travel ban, etc. In Figure 7 for The Monitor, we can see McAllen mayor, food bank, amid pandemic, school close, etc. We can also see the words jury trials as they were canceled because of the pandemic. Some cities or states, such as New Jersey, and New York, are absent in The Monitor, but as we can see, the four counties of the RGV are only present in The Monitor newspaper. It indicates that people who live in the RGV need to read local news outlets to get necessary updates. Simply reading the national newspaper will not meet the user’s information needs. Figure 5 shows the most frequent keyword identified by RAKE for USA Today. New York, Virus fear, small business, reopen America and keep distance are among the top key terms for USA Today. Nevertheless, Figure 8 shows that four counties and some major cities (Edinburg, McAllen) of the RGV are among the top keywords identified by RAKE for The Monitor. All these figures confirm that local newspaper coverage focus on events in the local areas, but national newspaper reports more from a broader view of what is happening in the whole country.

**Figure 9:**
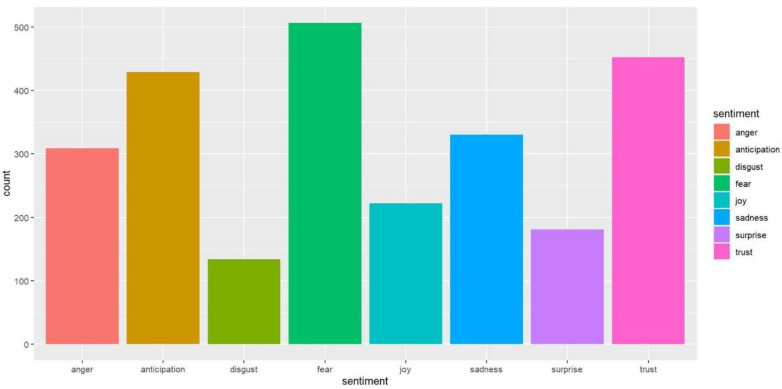
Number of words representing the sentiment dimensions (USA Today).

**Figure 10:**
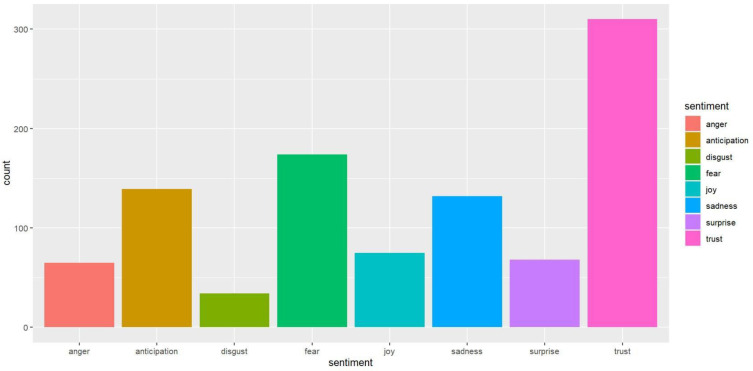
Number of words representing the sentiment dimensions (The Monitor).

Figure 9 and Figure 10 demonstrate which types of emotions are more prevalent in the newspapers. 

**Figure 11:**
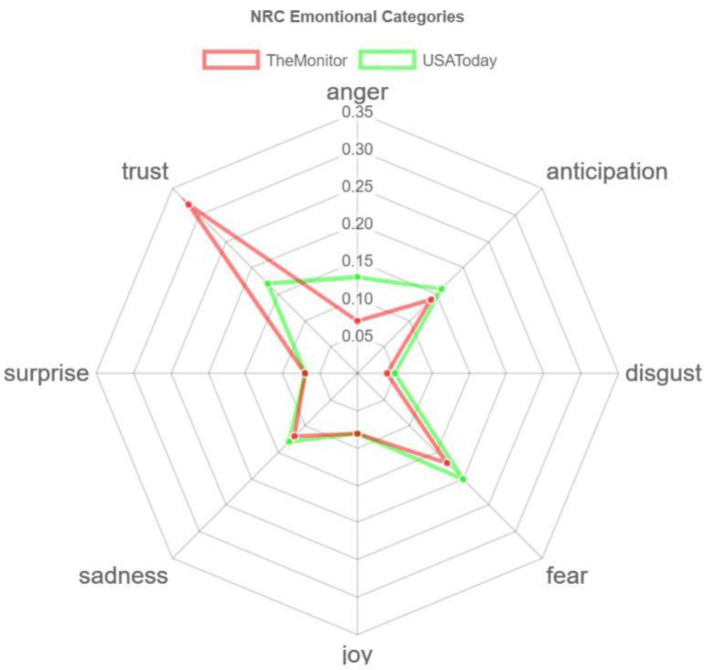
A radar chart of emotional valence.

Here, we can also see the contrasting results. While fear-related words are dominant in USA Today, for The Monitor, it is the trust emotion. Another interesting fact is that trust is the second emotion after fear in USA Today coverage, and they only have a slight difference. On the other hand, fear is the second emotion after trust in The Monitor. However, they have a significant difference. Moreover, the number of words that have anticipation is far more in USA Today than in The Monitor. However, the words that contain sadness and anger are also much more in USA Today compared to The Monitor. The radar chart in Figure 11 depicts this contrast more clearly. 

**Figure 12:**
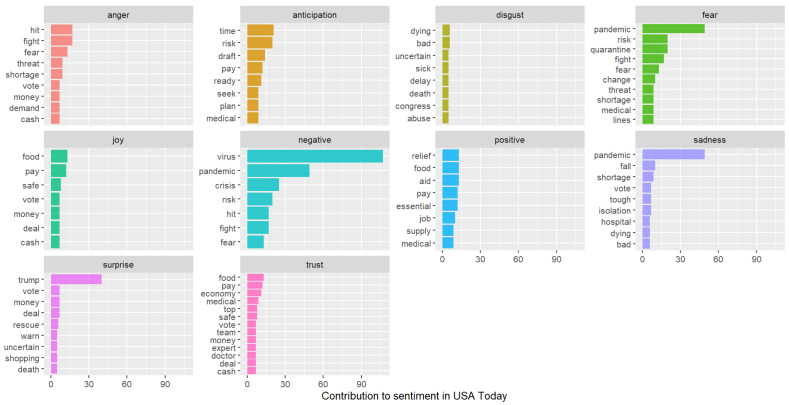
Top word distributions for each emotional valence (USA Today).

**Figure 13:**
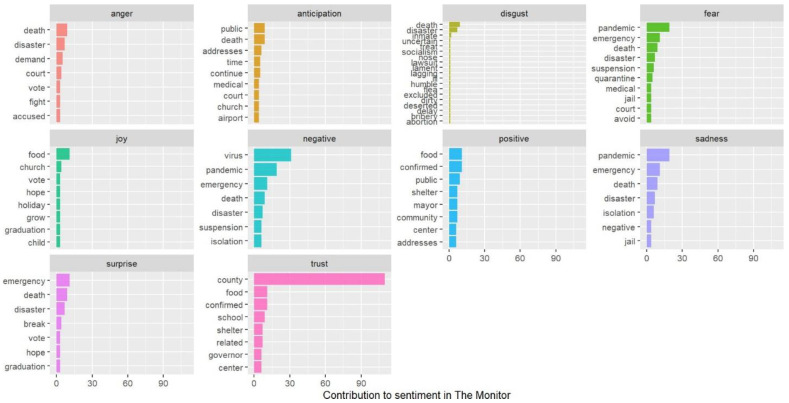
Top word distributions for each emotional valence (The Monitor).

The anger emotion is very high for USA Today compared to The Monitor. Trust is the dominant emotion for The Monitor, and there is a significant difference with USA Today. From Figure 12 and Figure 13, we can see the top word distributions for each emotion. 

The Monitor uses the word “county” most often in the news frames, and this word is absent in USA Today. For The Monitor, we can see the words public, shelter, mayor, community, and addresses are among some of the top words in positive emotion. For USA Today, relief, aid, pay, and supply are among the top words for positive emotion. In the fear emotion, we can see quarantine, fight, threat, shortage, etc., for USA Today. However, for The Monitor, emergency and suspension are some of the top words in fear emotion. Although these words evoke fear, the word choice is somewhat different for both newspapers.

## 5. Discussion

This study presents how newspapers play a crucial role in society by informing people about current issues and how they may affect them in their daily lives. In a health emergency like the COVID-19 pandemic, their significance becomes even more vital and imperative [73]. Since the beginning of the outbreak, newspapers have been a great source of information for the public on various topics, including identifying a novel coronavirus strain, the lockdown, masking and other prohibitions, governmental regulations, and details on developments of the COVID-19 vaccines. We can understand the COVID-19 pandemic well in this situation by analyzing emerging and extensively reported concerns, themes, and challenges and accompanying attitudes by analyzing the news headlines from both local and national perspectives.

This study demonstrates that the economic consequence frames are common in news headlines for both USA Today and The Monitor. We observe that supply chains and small businesses are common in the news frames. Major enterprises and investments have always been financially affected during a crisis [45]. The result could be both favorable and detrimental simultaneously, with some businesses making profits and others losing money. This financial impact [74] on local and national economies is referred to as the economic framing. As the disease spread, the political and economic aspects of the crisis gained momentum. Government travel advisories had an effect on certain businesses and economic sectors. Even though lifting the travel advisory permitted brief economic coverage, the health narrative is prioritized. Stakeholders during this phase provided reassurance and offered professional guidance that was crucial in this circumstance.

Moreover, our research illustrates the significance of strategic health communication. Strategic health communication refers to information about health issues that should come from specialists and disseminated by the media [75]. Health officials, the CDC, health workers, or health professionals frequently appeared in news headlines. The intention to immunize against COVID-19 was positively countered by authorities and specialists [75]. People who use information from these news sources that mention health experts have deep faith in them and are more likely to take preventive measures and get immunized. Another significant finding is that authorities had a significant presence in news frames. This is not surprising as reputable media frequently frames its news including attribution of responsibility [76,77]. National leaders were present in USA Today, whereas state and local authorities like the governor, judge, and mayor were present in The Monitor. According to literature, this frame of mentioning authorities is called attribution of responsibility. Coined by [77], it refers to tying responsibility for seeking answers or resolving issues to either the government or to people or an organization. From the newspaper headlines for USA Today and The Monitor, we identified the prevailing viewpoint amid the COVID-19 discussion. In doing so, we learned about COVID-19-related topics or issues frequently covered in the headlines of news publications. Through a triangulation of a co-occurrence network, RAKE, and sentiment analysis, this study offers fascinating insights about COVID-19 coverage in the news media, showing that while the subject or health topic may be the same, national, and local newspapers might report it in a different way.

To understand this complex reality, it is essential to be conscious of the various frames used during the period of medical emergency. As we can see, the pandemic highlights how important it is to read local newspapers because the headline frames of local newspapers vary significantly from those of national media. Contrary to prior pandemics, the overabundance of quasi-news articles in the mass media might cause a person’s confidence in health crisis journalism to waver, promoting the propagation of misconceptions. However, this study shows that journalists continue serving the public as messengers of information despite declining news consumption and trustworthiness. News media should report the facts/truths, no matter how depressing they may be [78]. The closures have made it harder for some communities to get local news, a condition known as “news deserts” [79]. Currently, more than one-fifth of Americans reside in such areas or areas that could soon develop into such news desert areas [79]. Communities suffer when local newspapers close. Because no one is covering the people or their tales [80], it raises concern if newspapers are closing. Hence, it is necessary to take appropriate steps in this regard.

Many people, including politicians, businesspeople, and academics, place a high value on comprehending topic evolution in big text collections [81]. However, it is challenging to examine why and how subjects change throughout time. The researchers must not only thoroughly analyze and distinguish between individual subjects but also pinpoint the crucial moments and factors that led to them, such as how new topics emerge, what sets them off or prevents them from developing, and how they progressively transform into other themes [81]. Therefore, when algorithms automatically detect the dominant topics from large volumes of news articles and visually present them for us to understand, they are valuable tools to analyze the newspapers. 

The findings of this research have several implications. Newspapers are tasked with preserving equilibrium in periods when content is quickly evolving [50] (e.g., whether or not masks are required for public safety). In regards of how much knowledge the common public can digest, there is also a lot to consider (particularly when the information is continually pessimistic), however, understanding epidemiology’s graphic presentations necessitate a high knowledge of numeracy. 

There’s also the difficulty of evaluating information (which can sometimes be intricate) and determining how it affects them, their families, their work, their daily routines, and so on. The newspapers have a responsibility to cover what’s happening in a way that is both useful and truthful, as well as to provide knowledge in a manner that is easy to understand, especially in a time of health emergency. Communicating in several formats—photos, infographics, first-person stories, news analysis—allows consumers to connect with the content in different ways while also assisting the news in striking that balance. The readers should be aware that, specifically throughout a pandemic, select daily newspapers will frequently share and update consumers with the most up-to-date information available. As a result, news organizations must realize that the pandemic may add to readers’ concern, skepticism, and, regrettably, the perception of fake news. 

By selecting the two most widely circulated newspapers in the U.S. national and local level, we can see the pandemic is covered from totally different angles. Although we can say authority responsibility frames are present in both newspapers, the focus is on diverse types of authorities. Both types of newspapers play an important role in informing the public and serving as a vital network between public health officials and the general people. Substantial pandemic coverage has ramifications for how people view the seriousness and effect of the public health issue and can have a significant impact on public actions.

Our analysis also demonstrates how information was constantly changing during this period. The public may find it challenging to properly comprehend and follow the best practices for prevention because of news coverage that includes frequently changing updates (daily figures of infection rate and death), as well as federal and state rules and recommendations. The audience relies largely on the news media for updates during times of seclusion and orders to stay at home, not only for statistics but also for information on societal norms and health policies.

Numerous articles concentrated on community mitigation tactics, like actions that the general public can take to stop the virus from spreading. This could mean that newspapers take on the additional role of social duty and public service by offering the most precise, reliable, and truthful information during a rapid world-changing catastrophe that causes public fear about economic and health challenges. Newspapers frequently employed public health terminology to characterize this epidemic, such as “quarantine,” “outbreak,” “epidemic,” and “pandemic,”. Although the initial reports almost entirely focused on the epidemiological scenario abroad, once the first cases started to show up in the United States, the number of articles emphasizing the outbreaks and measures of public health implemented in the US communities quickly rose.

## 6. Conclusions

Local newspapers’ front pages have more straightforward reporting that concentrates on the details of news events, while national newspapers are more prone to look at the larger picture, their coverage is more interpretative [82]. By applying a co-occurrence network, we can see the dominant frames for each newspaper. For instance, we can see the phrases CDC, scarcity, ventilator, mask, etc. for USA Today in frame 1. The virus dread, US political leaders, America, etc. are all included in frame 2. The governor and other local officials, including state, local, and other leaders, can be seen in frame 1 of The Monitor. Rio Grande Valley cities including Brownsville and Edinburg, and students, workers, and the community. Frame 3 includes the four RGV counties (Willacy, Starr, Hidalgo, and Cameron). RAKE’s top keywords for The Monitor included four RGV counties and a few large cities (Edinburg, McAllen). On the other side, RAKE found that the most often occurring keywords for USA Today were New York, virus fear, small business, reopen America, and keep distance. The sentiment analysis reveals that, whereas terms associated with dread predominate in USA Today, the sentiment for The Monitor is one of trust. Following faith in The Monitor, fear is the second feeling. For USA Today, we can associate the dread feeling with a quarantine, fight, threat, shortage, etc. However, according to The Monitor, words associated with fear include emergency and suspension. Despite the anxiety they inspire, both newspapers use different terms.

The COVID-19 pandemic continues, although a sizable amount of time has elapsed since it began. Considering this, we identified and analyzed the important thoughts and feelings expressed in COVID-19-related news headlines in our research. Our approach, and use of different methods shows that the United States and its national leaders, travel, economy, and sports are among the highest commonly stated issues in USA Today headlines. On the other hand, in the RGV and its four counties, local authorities, education, and local businesses are the main topics in The Monitor. The same topic, COVID-19, is covered differently in these two types of newspapers. Hence, it is important to focus on the whole picture during a health crisis. We will attempt to overcome the limitations of this research in subsequent works. 

## 7. Future Perspectives

To gather more comprehensive data, additional research might investigate both newspapers and television. Future research should look at if health crisis situations are politicized in news media or if news media coverage is generating vaccine hesitancy. Prospective studies should investigate the detrimental effects of health emergency news on young people’s mental health using longitudinal designs, reliable/valid measurements of news consumption, and ecological momentary assessments (examination of people’s daily thoughts and actions). Future research should take randomized controlled trials into account (For example, by distributing subscriptions to a sample of the public at random whenever a new WhatsApp service is launched to examine the psychological effects of distribution of health crisis news via WhatsApp).

The multi-class sentiment categorization and additional study on a comparison of all themes from local and national newspapers can be included in this. We can use topic modeling such as Latent Dirichlet Allocation (LDA) to pinpoint the primary topics of news headlines. Using a model like LSTM (Long short-term memory), which is a novel and intriguing technique for detecting emotion and sentiment, is also another route. LSTM recognizes the long-distance relationships between the words for determining the sentiment of a sentence [83]. In our upcoming research, we plan to study this model. Finally, to broaden the study’s focus and gain a comprehensive understanding of the US news media, we would like to include more newspapers from other US regions in our dataset.

## Data Availability

All news pieces analyzed in this research are available through the newspaper databases.
